# Supplementary material for: Efficient Mild Organosolv Lignin Extraction in a Flow-Through Setup Yielding Lignin with High β-O-4 Content
Source: Polymers (Basel). 2019 Nov 20;11(12):1913. doi: 10.3390/polym11121913 (PMC6960700; doi:10.3390/polym11121913)
Supplement: Supplementary file 1 [file polymers-11-01913-s001.pdf]

Efficient mild high β-O-4 organosolv lignin extraction in a flow setup yielding lignin with high β-O-4 content

Douwe S. Zijlstra[1], Coen A. Analbers[1], Joren de Korte[1], Erwin Wilbers[1] and Peter J. Deuss[1,*]

[1] Department of Chemical Engineering (ENTEG), University of Groningen, Nijenborgh 4, 9747 AG Groningen, The Netherlands
* Correspondence: p.j.deuss@rug.nl

1. Methodologies

Yield correction for alcohol incorporation

To compensate the yield for the alcohol incorporation into the lignin, a correction factor is calculated. First the mass of the different monolignols is determined.

	No incorporation	EtOH	nPrOH	nBuOH
S	226	254	268	282
G	196	224	238	252
H	166	194	208	222

Table S1: Mass of the monolignols with solvent incorporation.

For every monolignol an individual correction factor needs to be calculated. This is the relative mass of the lignin that belongs to the incorporated solvent, for example for a S unit with EtOH incorporation.

$$Correction\ factor\ S = \frac{\mathrm{Mass\ (S\ \beta' - O - 4)}}{\mathrm{Mass\ (S\ \beta - O - 4)}} - 1\ =\ \frac{254}{226} - 1\ =\ 0.124$$

	EtOH	nPrOH	nBuOH
S	0.124	0.186	0.248
G	0.143	0.214	0.286
H	0.169	0.253	0.337

Table S2: Correction factors for determination of the corrected yield.

Together with the fractions S, G and H, the corrected yield can be calculated.
For example, for a lignin fraction extracted with EtOH:

$$Corrected\ yield\ (mg) =$$
$$Experimental\ yield\ (mg) * (1 - \frac{\beta' - O - 4}{100} * (0.124 * fraction\ S + 0.143 * fraction\ G + 0.169 * fraction\ H))$$

Calculations of average value

$$Average\ value = \frac{mass\ of\ fraction\ 1 * corresponding\ value\ 1 + mass\ of\ frac\ 2 * corr\ value + \ ...}{Sum\ of\ the\ total\ mass\ of\ the\ fractions}$$

Formula has been applied to calculate the average condensation, β-O-4 content, H/G/S ratio and molecular weight.

Data representation

All the graphs are made with OriginPro 8.5. All data fits for the extraction efficiency are done with nonlinear curve fitting, as are most of the data fits for the β-O-4 content over time. If an insufficient fit was obtained a second order polynomial fit was used for fitting.

Flow-through setup

The flow-through system consist of a glass solvent reservoir (800mL), pneumatic oscillatory pump (Williams; P250V225), pressure indicator (0-400 bar), GC oven (HP, 5890 series II), reactor (100 mL, cylindrical (length: 20 cm, diameter: 2,6 cm), SS 316L), Type K thermocouple with indicator, particulate filter (Swagelok, ¼ in. tube fitting, 2 micron pore size), adjustable back pressure regulator (Gommer B.V., BP-3 series, SS 316L) with pressure indicator (0-40 bar) and precision balance (Kern, PCB 2500-2). These units are connected by tubes and fittings (Swagelok, SS-316L, ½ and ¼ in.). Both openings of the reactor are closed with a glass filter (custom made, sintered, P2).

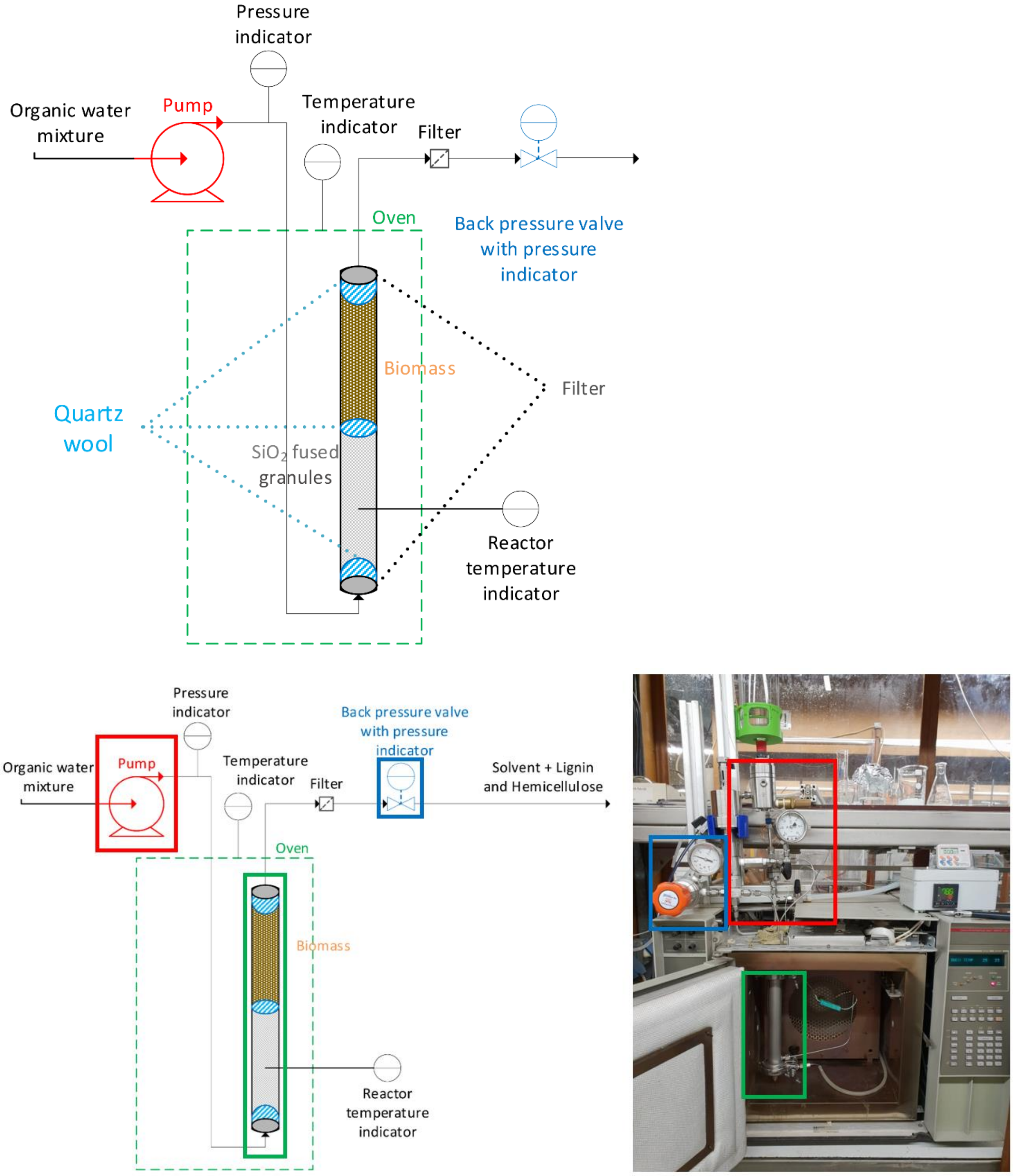

Figure S1. Schematic representation of the developed flow-through setup (**top**) and a picture of the setup with the most important features highlighted (**bottom**).

Extraction efficiency and β-O-4 content vs Solvent used

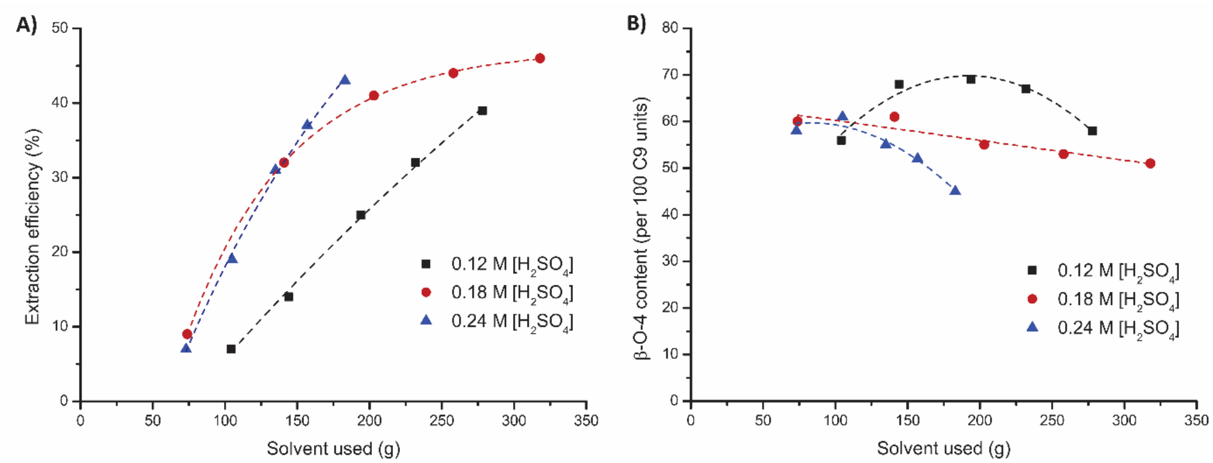

Figure S2. Influence of the different [H_2SO_4] concentrations on the mild organosolv extraction, (80:20 EtOH/H_2O, 120 ˚C, 5 hours) on **A)** extraction efficiency (corrected for alcohol incorporation as determined by 2D HSQC) and **B)** total β-O-4 content as determined by 2D HSQC, plotted against solvent used.

Extraction results

Time (hours)	Yield (mg)	Extraction efficiency (%)[a,b]	Solvent used (g)[b]	Solvent efficiency (mg/g)	H/G/S ratio (%)	Cond.[c]	Total β-O-4[d]	β-O-4[d]	β'-O-4[d]	Mn (Da)[e]	Mw (Da)[e]
1	1157	13	69	17	8/44/48	0	61	28	33	874	1530
2	1691	31	138	25	2/34/64	0	66	24	42	982	2230
3	1103	43	206	16	1/31/68	2.5	60	17	43	1080	2690
4	585	49	272	9	0/31/69	4	62	16	45	1110	2970
5	268	52	339	4	0/31/69	5	55	16	38	1060	2730

Table S1. Extraction efficiency and lignin properties of Entry **1** (EtOH/H_2O 80:20, 0.18 M [H_2SO_4]. [a] Corrected for alcohol incorporation as determined by 2D-HSQC, [b] Cumulative values, [c] expressed as percentage $S_{condensed}$ of the total S content [d] linking motifs per 100 C9 units as determined by 2D-HSQC (1.3 correction factor applied), [e] determined by GPC (THF).

Time (hours)	Yield (mg)	Extraction efficiency (%)[a,b]	Solvent used (g)[b]	Solvent efficiency (mg/g)	H/G/S ratio (%)	Cond.[c]	Total β-O-4[d]	β-O-4[d]	β'-O-4[d]	Mn (Da)[e]	Mw (Da)[e]
1	663	7	104	6	7/49/44	0	56	38	18	1100	1920
2	668	14	144	16	3/40/57	1	68	28	40	1320	2390
3	1073	25	194	22	1/35/64	0	69	25	44	1420	2970
4	684	32	232	18	0/32/68	1	67	24	43	1500	3560
5	711	39	278	15	0/31/69	3	58	17	41	1730	4280

Table S2. Extraction efficiency and lignin properties of Entry **2** (EtOH/H_2O 80:20, 0.12 M [H_2SO_4] *Different walnut source. [a] Corrected for alcohol incorporation as determined by 2D-HSQC, [b] Cumulative values, [c] expressed as percentage $S_{condensed}$ of the total S content [d] linking motifs per 100 C9 units as determined by 2D-HSQC (1.3 correction factor applied), [e] determined by GPC (THF).

Time (hours)	Yield (mg)	Extraction efficiency (%)[a,b]	Solvent used (g)[b]	Solvent efficiency (mg/g)	H/G/S ratio (%)	Cond.[c]	Total β-O-4[d]	β-O-4[d]	β'-O-4[d]	Mn (Da)[e]	Mw (Da)[e]
1	829	9	74	11	6/45/49	2	60	27	33	1250	2300
2	2078	32	141	31	1/34/65	2	61	18	42	1390	3660
3	892	41	203	14	0/32/68	4	55	18	36	1580	4580
4	292	44	258	5	0/32/68	5.5	53	12	41	1680	4790
5	159	46	318	3	0/31/69	10	51	14	37	1870	5720

Table S3. Extraction efficiency and lignin properties of Entry **3** (EtOH/H_2O 80:20, 0.18 M [H_2SO_4] *Different walnut source. [a] Corrected for alcohol incorporation as determined by 2D-HSQC, [b] Cumulative values, [c] expressed as percentage $S_{condensed}$ of the total S content [d] linking motifs per 100 C9 units as determined by 2D-HSQC (1.3 correction factor applied), [e] determined by GPC (THF).

Time (hours)	Yield (mg)	Extraction efficiency (%)[a,b]	Solvent used (g)[b]	Solvent efficiency (mg/g)	H/G/S ratio (%)	Cond.[c]	Total β-O-4[d]	β-O-4[d]	β'-O-4[d]	Mn (Da)[e]	Mw (Da)[e]
1	672	7	73	9	8/47/45	0	58	33	25	980	1680
2	1050	19	105	33	1/39/60	0	61	19	42	1180	2740
3	1133	31	135	38	1/31/68	7	55	14	41	1510	3680
4	575	37	157	26	0/30/70	7	52	12	40	1600	4130
5	501	43	183	19	0/30/70	10.5	45	8	37	1510	3900

Table S4. Extraction efficiency and lignin properties of Entry **4** (EtOH/H_2O 80:20, 0.24 M [H_2SO_4] *Different walnut source. [a] Corrected for alcohol incorporation as determined by 2D-HSQC, [b] Cumulative values, [c] expressed as percentage $S_{condensed}$ of the total S content [d] linking motifs per 100 C9 units as determined by 2D-HSQC (1.3 correction factor applied), [e] determined by GPC (THF).

Time (hours)	Yield (mg)	Extraction efficiency (%)[a,b]	Solvent used (g)[b]	Solvent efficiency (mg/g)	H/G/S ratio (%)	Cond.[c]	Total β-O-4[d]	β-O-4[d]	β'-O-4[d]	Mn (Da)[e]	Mw (Da)[e]
1	729	8	87	8	7/44/49	0	51	27	24	961	1800
2	1373	23	141	25	2/32/66	2.5	55	27	28	783	1600
3	1027	34	196	19	0/31/69	5	48	22	25	804	1680
4	181	36	231	5	0/33/67	6.5	51	22	29	815	1880

Table S5. Extraction efficiency and lignin properties of Entry **5** (EtOH/H_2O 50:50, 0.18 M [H_2SO_4] [a] Corrected for alcohol incorporation as determined by 2D-HSQC, [b] Cumulative values, [c] expressed as percentage $S_{condensed}$ of the total S content [d] linking motifs per 100 C9 units as determined by 2D-HSQC (1.3 correction factor applied), [e] determined by GPC (THF).

Time (hours)	Yield (mg)	Extraction efficiency (%)[a,b]	Solvent used (g)[b]	Solvent efficiency (mg/g)	H/G/S ratio (%)	Cond.[c]	Total β-O-4[d]	β-O-4[d]	β'-O-4[d]	Mn (Da)[e]	Mw (Da)[e]
1	1046	11	55	19	4/46/50	0	62	10	52	1130	2520
2	2197	35	114	37	1/31/68	9.5	53	5	48	1320	3710
3	1360	50	172	23	0/23/77	20.5	32	0	32	1150	3280
4	528	56	231	9	0/31/69	22	9	0	9	1160	3040
5	141	57	290	2	0/28/72	25.5	11	0	11	1210	3050

Table S6. Extraction efficiency and lignin properties of Entry **6** (EtOH/H_2O 95:5, 0.18 M [H_2SO_4] [a] Corrected for alcohol incorporation as determined by 2D-HSQC, [b] Cumulative values, [c] expressed as percentage $S_{condensed}$ of the total S content [d] linking motifs per 100 C9 units as determined by 2D-HSQC (1.3 correction factor applied), [e] determined by GPC (THF).

Time (hours)	Yield (mg)	Extraction efficiency (%)[a,b]	Solvent used (g)[b]	Solvent efficiency (mg/g)	H/G/S ratio (%)	Cond.[c]	Total β-O-4[d]	β-O-4[d]	β'-O-4[d]	Mn (Da)[e]	Mw (Da)[e]
1	1095	12	84	13	7/44/49	0	58	9	49	1030	2290
2	1991	33	147	31	3/33/64	0	72	19	53	1110	2660
3	1071	45	200	20	1/30/69	0	65	11	53	1390	4690
4	726	53	258	13	1/30/69	2.5	64	9	55	1460	5810
5	400	57	321	6	0/27/73	2	66	7	59	1400	5690

Table S7. Extraction efficiency and lignin properties of Entry **7** (nPrOH/H_2O 80:20, 0.18 M [H_2SO_4] [a] Corrected for alcohol incorporation as determined by 2D-HSQC, [b] Cumulative values, [c] expressed as percentage $S_{condensed}$ of the total S content [d] linking motifs per 100 C9 units as determined by 2D-HSQC (1.3 correction factor applied), [e] determined by GPC (THF).

Time (hours)	Yield (mg)	Extraction efficiency (%)[a,b]	Solvent used (g)[b]	Solvent efficiency (mg/g)	H/G/S ratio (%)	Cond.[c]	Total β-O-4[d]	β-O-4[d]	β'-O-4[d]	Mn (Da)[e]	Mw (Da)[e]
1	1926	21	59	33	4/39/57	0	68	5	63	1310	4500
2	2472	48	113	45	0/25/75	8.5	57	2	55	1400	6650
3	2260	72	207	24	0/27/73	20	41	0	41	1320	5440
4	132	74	253	3	0/32/68	17	32	0	32	1360	4980

Table S8. Extraction efficiency and lignin properties of Entry **8** (nBuOH/H_2O/1,4-dioxane 80:15:5, 0.18 M [H_2SO_4] [a] Corrected for alcohol incorporation as determined by 2D-HSQC, [b] Cumulative values, [c] expressed as percentage $S_{condensed}$ of the total S content [d] linking motifs per 100 C9 units as determined by 2D-HSQC (1.3 correction factor applied), [e] determined by GPC (THF).

Time (hours)	Yield (mg)	Extraction efficiency (%)[a,b]	Solvent used (g)[b]	Solvent efficiency (mg/g)	H/G/S ratio (%)	Cond.[c]	Total β-O-4[d]	β-O-4[d]	β'-O-4[d]	Mn (Da)[e]	Mw (Da)[e]
1	1860	19	58	32	6/41/53	0	58	20	38	1130	2061
2	2010	39	125	30	2/32/66	6.5	55	14	41	1379	3042
3	950	48	185	16	0/32/68	10.5	53	12	41	1365	2963
4	380	52	242	7	0/32/68	11.5	50	10	40	1413	3200
5	100	53	301	2	0/26/74	13.5	43	10	33	1408	3250

Table S9. Extraction efficiency and lignin properties of Entry **9** (nEtOH/H_2O/1,4-dioxane 80:15:5, 0.18 M [H_2SO_4] [a] Corrected for alcohol incorporation as determined by 2D-HSQC, [b] Cumulative values, [c] expressed as percentage $S_{condensed}$ of the total S content [d] linking motifs per 100 C9 units as determined by 2D-HSQC (1.3 correction factor applied), [e] determined by GPC (THF).

Time (hours)	Yield (mg)	Extraction efficiency (%)[a,b]	Solvent used (g)[b]	Solvent efficiency (mg/g)	H/G/S ratio (%)	Cond.[c]	Total β-O-4[d]	β-O-4[d]	β'-O-4[d]	Mn (Da)[e]	Mw (Da)[e]
1	1165	12	44	26	5/42/53	0	63	19	44	1138	2157
2	2581	35	95	51	1/33/66	9.5	59	14	45	1351	3224
3	1402	52	148	26	0/32/68	18.5	46	10	36	1339	3302
4	516	58	203	9	0/26/74	24	42	4	38	1374	3479
5	110	59	247	3	0/27/73	24.5	39	4	35	1295	3032

Table S10. Extraction efficiency and lignin properties of Entry **10** (nPrOH/H_2O/1,4-dioxane 80:15:5, 0.18 M [H_2SO_4] [a] Corrected for alcohol incorporation as determined by 2D-HSQC, [b] Cumulative values, [c] expressed as percentage $S_{condensed}$ of the total S content [d] linking motifs per 100 C9 units as determined by 2D-HSQC (1.3 correction factor applied), [e] determined by GPC (THF).

Time (hours)	Yield (mg)	Extraction efficiency (%)[a,b]	Solvent used (g)[b]	Solvent efficiency (mg/g)	H/G/S ratio (%)	Cond.[c]	Total β-O-4[d]	β-O-4[d]	β'-O-4[d]	Mn (Da)[e]	Mw (Da)[e]
0.5	179	1	28	6	15/49/36	0	59	21	38	778	1350
1	2116	12	61	64	8/44/48	0	62	13	49	962	1760
1.5	4498	37	86	179	2/32/66	6	59	6	53	1180	2950
2	3920	58	129	92	0/30/70	15	40	3	37	1190	3350
2.5	1623	67	164	46	0/36/64	14	36	1	35	1170	3200
3	764	71	194	26	0/32/68	17.5	31	0	31	1170	5990
3.5	469	74	215	22	0/29/71	18	30	0	30	1160	2950
4	661	77	247	20	0/36/64	16	18	0	18	1120	2740
4.5	517	80	279	17	0/34/66	18	15	0	15	1100	2730
5	452	82	313	23	0/32/68	18	12	0	12	1080	2650

Table S11. Extraction efficiency and lignin properties of Entry **11** (EtOH/H_2O 95:5, 0.18 M [H_2SO_4], 40 gram loading. [a] Corrected for alcohol incorporation as determined by 2D-HSQC, [b] Cumulative values, [c] expressed as percentage $S_{condensed}$ of the total S content [d] linking motifs per 100 C9 units as determined by 2D-HSQC (1.3 correction factor applied), [e] determined by GPC (THF).

Time (hours)	Yield (mg)	Extraction efficiency (%)[a,b]	Solvent used (g)[b]	Solvent efficiency (mg/g)	H/G/S ratio (%)	Cond.[c]	Total β-O-4[d]	β-O-4[d]	β'-O-4[d]	Mn (Da)[e]	Mw (Da)[e]
1	246	3	62	4	0/85/15	0	58	10	48	1090	2590
2	570	11	117	10	0/82/18	0	66	5	61	1410	3670
3	392	17	169	8	0/82/18	0	53	4	49	1370	3580
4	189	19	229	3	0/88/12	0	44	4	40	1340	3360
5	72	20	290	1	0/100/0	0	37	2	35	1350	3310

Table S12. Extraction efficiency and lignin properties of Entry **12** (EtOH/H_2O 95:5, 0.18 M [H_2SO_4], Spruce/poplar. [a] Corrected for alcohol incorporation as determined by 2D-HSQC, [b] Cumulative values, [c] expressed as percentage $S_{condensed}$ of the total S content [d] linking motifs per 100 C9 units as determined by 2D-HSQC (1.3 correction factor applied), [e] determined by GPC (THF).

Time (hours)	Yield (mg)	Extraction efficiency (%)[a,b]	Solvent used (g)[b]	Solvent efficiency (mg/g)	H/G/S ratio (%)	Cond.[c]	Total β-O-4[d]	β-O-4[d]	β'-O-4[d]	Mn (Da)[e]	Mw (Da)[e]
1	896	9	57	16	0/100/0	35	25	0	25	912	2140
2	963	18	112	18	0/100/0	0	60	0	60	1240	3230
3	579	24	178	9	0/100/0	25	38	0	38	1330	3420
4	338	27	236	6	0/100/0	100	0	0	0	1360	3260
5	147	29	292	3	0/100/0	100	6	0	6	1330	2810

Table S13. Extraction efficiency and lignin properties of Entry **13** (EtOH/H_2O 95:5, 0.18 M [H_2SO_4], Cedar wood. [a] Corrected for alcohol incorporation as determined by 2D-HSQC, [b] Cumulative values, [c] expressed as percentage $S_{condensed}$ of the total S content [d] linking motifs per 100 C9 units as determined by 2D-HSQC (1.3 correction factor applied), [e] determined by GPC (THF).

Time (hours)	Yield (mg)	Extraction efficiency (%)[a,b]	Solvent used (g)[b]	Solvent efficiency (mg/g)	H/G/S ratio (%)	Cond.[c]	Total β-O-4[d]	β-O-4[d]	β'-O-4[d]	Mn (Da)[e]	Mw (Da)[e]
1	212	4	50	4	0/37/63	0	60	16	44	1380	3090
2	1118	22	106	20	0/19/81	10	57	9	48	1510	3950
3	678	33	162	12	0/14/86	22	38	1	37	1290	3060
4	464	41	218	8	0/15/85	30	19	0	19	1170	2620
5	171	44	274	3	0/12/88	42	13	0	13	1120	2380

Table S14. Extraction efficiency and lignin properties of Entry **14** (EtOH/H_2O 95:5, 0.18 M [H_2SO_4], Beech wood). [a] Corrected for alcohol incorporation as determined by 2D-HSQC, [b] Cumulative values, [c] expressed as percentage $S_{condensed}$ of the total S content [d] linking motifs per 100 C9 units as determined by 2D-HSQC (1.3 correction factor applied), [e] determined by GPC (THF).

Entry	Yield (mg)	Extraction efficiency (%)[a,b]	Solvent used (g)[b]	Solvent efficiency (mg/g)	H/G/S ratio (%)	Cond.[c]	Total β-O-4[d]	β-O-4[d]	β'-O-4[d]	Mn (Da)[e]	Mw (Da)[e]
**15**	2112	46	83	25	0/36/64	13	28	8	20	978	2450
**16**	1760	38	90	20	0/35/65	11	38	16	22	1180	2610
**17**	1832	40	80	23	0/42/58	24	10	0	10	968	2070
**18**	1777	39	84	21	3/36/61	2	60	9	51	1140	3360
**19**	1985	43	83	24	1/34/65	12	44	2	42	1220	3800
**20**	320	9	80	4	0/84/16	12	30	2	28	982	2280
**21**	1294	28	80	16	0/100/0	16	8	1	7	971	2160
**22**	586	19	80	8	0/18/82	27	26	0	26	958	2000

Table S15. Extraction efficiency and lignin properties of all batch experiments. [a] Corrected for alcohol incorporation as determined by 2D-HSQC, [b] Cumulative values, [c] expressed as percentage $S_{condensed}$ of the total S content [d] linking motifs per 100 C9 units as determined by 2D-HSQC (1.3 correction factor applied), [e] determined by GPC (THF).

Entry 15: Walnut, 80:20 EtOH/H_2O, 0.18 M [H_2SO_4], 120 ˚C, 5 hours
Entry 16: Walnut, 50:50 EtOH/H_2O, 0.18 M [H_2SO_4], 120 ˚C, 5 hours
Entry 17: Walnut, 95:5 EtOH/H_2O, 0.18 M [H_2SO_4], 120 ˚C, 5 hours
Entry 18: Walnut, 80:20 nPrOH/H_2O, 0.18 M [H_2SO_4], 120 ˚C, 5 hours
Entry 19: Walnut, 80:15:5 nBuOH/H_2O/1,4-dioxane, 0.18 M [H_2SO_4], 120 ˚C, 5 hours
Entry 20: Pre-paper, 80:20 EtOH/H_2O, 0.18 M [H_2SO_4], 120 ˚C, 5 hours
Entry 21: Cedar wood, 80:20 EtOH/H_2O, 0.18 M [H_2SO_4], 120 ˚C, 5 hours
Entry 22: Beech wood, 80:20 EtOH/H_2O, 0.18 M [H_2SO_4], 120 ˚C, 5 hours

Reproducibility

Time (hours)	Yield (mg)	Extraction efficiency (%)[a,b]	Solvent used (g)[b]	Solvent efficiency (mg/g)	H/G/S ratio (%)	Cond.[c]	Total β-O-4[d]	β-O-4[d]	β'-O-4[d]	Mn (Da)[e]	Mw (Da)[e]
1	1157	13	69	17	8/44/48	0	61	28	33	874	1530
2	1691	31	138	25	2/34/64	0	66	24	42	982	2230
3	1103	43	206	16	1/31/68	2.5	60	17	43	1080	2690
4	585	49	272	9	0/31/69	4	62	16	45	1110	2970
5	268	52	339	4	0/31/69	5	55	16	38	1060	2730

Table S16. Extraction efficiency and lignin properties of Entry **1** in appendix A (EtOH/H_2O 80:20, 0.18 M [H_2SO_4]). [a] Corrected for alcohol incorporation as determined by 2D-HSQC, [b] Cumulative values, [c] expressed as percentage $S_{condensed}$ of the total S content [d] linking motifs per 100 C9 units as determined by 2D-HSQC (1.3 correction factor applied), [e] determined by GPC (THF).

Time (hours)	Yield (mg)	Extraction efficiency (%)[a,b]	Solvent used (g)[b]	Solvent efficiency (mg/g)	H/G/S ratio (%)	Cond.[c]	Total β-O-4[d]	β-O-4[d]	β'-O-4[d]	Mn (Da)[e]	Mw (Da)[e]
1	1304	16	52	25	6/41/53	0	62	21	42	921	1940
2	1782	37	108	32	0/33/67	1	63	18	45	1090	2840
3	907	48	166	16	0/31/69	8	49	14	35	1140	3280
4	288	52	226	5	0/31/69	9.5	48	12	35	1280	3640
5	39	52	285	1	0/34/66	7.5	44	12	32	1050	3340

Table S17. Extraction efficiency and lignin properties of a redo of extraction **1** (EtOH/H_2O 80:20, 0.18 M [H_2SO_4]). [a] Corrected for alcohol incorporation as determined by 2D-HSQC, [b] Cumulative values, [c] expressed as percentage $S_{condensed}$ of the total S content [d] linking motifs per 100 C9 units as determined by 2D-HSQC (1.3 correction factor applied), [e] determined by GPC (THF).
